# Cell Blood Count Alterations and Patterns of Anaemia in Autoimmune Atrophic Gastritis at Diagnosis: A Multicentre Study

**DOI:** 10.3390/jcm8111992

**Published:** 2019-11-15

**Authors:** Marco Vincenzo Lenti, Edith Lahner, Gaetano Bergamaschi, Emanuela Miceli, Laura Conti, Sara Massironi, Sara Cococcia, Alessandra Zilli, Flavio Caprioli, Maurizio Vecchi, Stefania Maiero, Renato Cannizzaro, Gino Roberto Corazza, Bruno Annibale, Antonio Di Sabatino

**Affiliations:** 1 First Department of Medicine, Istituto di Ricovero e Cura a Carattere Scientifico San Matteo Hospital Foundation, University of Pavia, Piazzale Golgi 19, 27100 Pavia, Italyn.bergamaschi@smatteo.pv.it (G.B.); e.miceli@smatteo.pv.it (E.M.); sara.cococcia@gmail.com (S.C.); gr.corazza@smatteo.pv.it (G.R.C.); 2 Department of Surgical-Medical Sciences and Translational Medicine, Digestive and Liver Disease Unit, Sant’Andrea Hospital, Sapienza University of Rome, 00185 Roma, Italy; edith.lahner@uniroma1.it (E.L.); lau88conti@gmail.com (L.C.); bruno.annibale@uniroma1.it (B.A.); 3 Department of Gastroenterology and Endoscopy, IRCCS Ca’ Granda Foundation, University of Milan, 20122 Milano, Italy; sara.massironi@policlinico.mi.it (S.M.); alessandra.zilli86@gmail.com (A.Z.); flavio.caprioli@unimi.it (F.C.); maurizio.vecchi@unimi.it (M.V.); 4 Oncological Gastroenterology Unit, Centro di Riferimento Oncologico di Aviano (CRO) IRCCS, 33081 Aviano, Italy; smaiero@cro.it (S.M.);

**Keywords:** anisocytosis, iron deficiency, pernicious anaemia, vitamin B12

## Abstract

Background: Autoimmune atrophic gastritis (AAG) leads to iron and/or vitamin B12 malabsorption, with subsequent haematological alterations which could represent the sole clinical manifestation. We aimed to assess patterns of anaemia and micronutrient deficiencies in patients with AAG at the time of diagnosis. Methods: Observational, multicentre, cross-sectional study including consecutive adult patients diagnosed with AAG within the last ten years. Cell blood count, red cell distribution width, serum vitamin B12, and ferritin were collected. Multivariate analysis for predictive factors of anaemia was computed. Results: 654 AAG patients (mean age 59.2 ± 13.8 years, female (F): male (M) ratio = 2.3:1) were included. Anaemia was present in 316 patients (48.3%; mean age 60.1 ± 15.8 years, F:M ratio = 2.3:1). Pernicious anaemia (132/316 cases, 41.7%) was more common in males (27.1% versus 12.4%; *p* = 0.001) and in older patients (63.0 ± 14.6 versus 58.9 ± 14.9 years; *p* = 0.014), while iron deficiency anaemia (112/316 cases, 35.4%) was more common in females (16.9% versus 10.0%; *p* = 0.039) and in younger patients (56.8 ± 16.6 versus 60.2 ± 14.6 years; *p* = 0.043). The prevalence of iron deficiency was equally distributed between anaemic and non-anaemic patients (*p* = 0.9). Anisocytosis (odds ratio: 10.65, 95% confidence interval: 6.13–18.50, *p* < 0.0001) was independently associated with anaemia. Conclusions: Anaemia is a common manifestation in AAG patients, mostly due to micronutrient deficiencies. Scant haematologic alterations and micronutrient deficiencies may precede overt anaemia.

## 1. Introduction

Autoimmune atrophic gastritis (AAG) is an organ-specific, immune-mediated disorder that affects the corpus and fundus of the stomach, causing atrophy of the oxyntic mucosa, impaired gastric acid secretion, and intrinsic factor deficiency [1,2]. AAG is a relatively frequent condition, particularly in elderly individuals, even if any age group may be affected [3,4,5]. In the natural history of AAG, malabsorption of micronutrients, especially vitamin B12 and iron, occurs over time [6,7]. Micronutrient deficiencies lead to various manifestations, in particular, red blood cell alterations, which may represent the sole clinical presentation of AAG [4,5,6,7,8]. A diagnosis of AAG can be easily made in case of overt pernicious anaemia [9,10]. However, according to a previous study of ours [4], a considerable proportion of AAG patients have scant and subtle haematological alterations, including isolated mean corpuscular volume (MCV) changes and isolated increase of red cell distribution width (RDW). A high proportion of AAG patients may also suffer from iron deficiency anaemia long before the onset of vitamin B12 deficiency, or may present a dimorphic anaemia as a consequence of combined deficiency [6]. According to previous observational studies, the prevalence of anaemia in patients with AAG ranges from 19% to 94% (Table 1) [4,6,11,12,13,14,15,16,17,18]. However, despite the relevant clinical burden, ad hoc studies focusing on red blood cell alterations in large cohorts of patients with histologically-confirmed AAG are lacking. As we have recently shown, haematological alterations might be overlooked, especially in female patients and in non-haematological settings, determining substantial AAG diagnostic delay [18,19]. 

On these bases, the primary aim of this cross-sectional study was to assess the prevalence and types of anaemia and micronutrient deficiencies, according to age and gender, in AAG patients at the time of diagnosis. The secondary aim was to identify patterns of red blood cell alterations and putative predictors of anaemia. Lastly, recovery from anaemia at a one-year follow-up was evaluated in a subgroup of AAG patients. 

## 2. Materials and Methods

### 2.1. Participating Centres, Patient Selection, and Definition of Anaemia

Four Italian, tertiary referral centres for the diagnosis and management of AAG participated in this study (Istituto di Ricovero e Cura a Carattere Scientifico San Matteo Hospital in Pavia, Sant’Andrea Hospital in Rome, IRCCS Ca’ Granda Hospital in Milan, and IRCCS National Cancer Institute in Aviano). In these centres, most Italian adult AAG patients are referred and followed-up. Each centre has a dedicated database in which relevant data, including sociodemographic characteristics and medical history, have been prospectively collected from all consecutive adult AAG patients over the last ten years. According to internationally agreed criteria, AAG diagnosis was based on histological grounds following the updated Sydney–Houston criteria [20]. The presence of AAG-related serum antibodies, namely anti-parietal cell antibodies (PCA) and anti-intrinsic factor antibodies, was not considered a necessary feature in case of clear and undoubted histological lesions [21]. In fact, these antibodies have no absolute accuracy for AAG, and may not be present in late disease stage [12,22,23] In all cases, gastric biopsy specimens were reviewed from expert gastrointestinal pathologists. Histopathological alterations consistent with any stage of AAG include: (i) atrophy of gastric oxyntic mucosa, (ii) absence of atrophy in gastric antrum mucosa, (iii) concurrent evidence of extensive intestinal and/or pseudopyloric metaplasia, and (iv) hyperplasia of gastrin-producing cells and hyperplasia of enterochromaffin-like cells. Patients with uncertain AAG diagnosis (e.g., patchy or uncertain mucosal lesions), active H. pylori infection, atrophic pangastritis, and with incomplete medical history, were not included in the study. All data from adult (≥18 years old) AAG patients were anonymised and collated onto a predefined spreadsheet. All queries regarding uncertain data were resolved via email or meetings through consensus with the study coordinators (MVL, EL). Particularly, demographic and clinical data from patients’ medical records were collected and analysed, including gender, age, main clinical presentation, comorbidities, and histopathological features according to Operative Link on Gastritis Assessment (OLGA) and Operative Link on Gastric Intestinal Metaplasia Assessment (OLGIM) [24,25]. Relevant laboratory data at the time of AAG diagnosis (±1 month) were collected, including haemoglobin, MCV (normal range 80–98 femtoliter), RDW (normal range 11–15%), platelets (normal range 150,000–450,000/microliter), serum vitamin B12 (deficient if < 200 ng/L), iron (deficient if < 55 ng/mL), ferritin (deficient if < 30 ng/mL), folate (deficient if < 4 ng/mL), homocysteine (increased if > 12 µmol/L), and presence or absence of serum PCA [26]. Complete blood counts were performed by a Cell-Dyn Sapphire. Vitamin B12 was assessed in serum by an automated immunochemistry analyser, which is a solid-phase, competitive chemiluminescent enzyme immunoassay. PCA were detected by either immunofluorescence or enzyme-linked immunosorbent assay (ELISA) techniques. Iron, ferritin, and folate were detected by a colorimetric assay. Homocysteine was assessed with a fluorometric assay kit in plasma or serum. Anaemia was classified according to the World Health Organisation (WHO), i.e., haemoglobin < 120 g/L in females and < 130 g/L in males living at sea level [27]. Transferrin, reticulocytes, inflammatory markers (e.g., C reactive protein), and urine methylmalonic acid were not included in the final analyses, as they were missing in many patients. Iron deficiency anaemia was defined as the presence of anaemia and low iron and ferritin levels, while pernicious anaemia was defined as the presence of macrocytic anaemia (or normocytic in case of dimorphic anaemia) and vitamin B12 and/or folate deficiency. Anaemia of chronic disease was inferred in case of ferritin > 100 ng/mL and iron < 55 ng/mL. A few patients with concomitant haematological disorders were excluded a priori, as it was not possible to ascertain the aetiology of red blood cell alterations. In a subgroup of 181 patients belonging to the Pavia cohort, for whom cell blood count was available after 12 months since diagnosis, one-year follow-up data were reported. The study was approved by each local Ethics Committee and all patients gave their informed consent for the anonymised publication of data. The results of this study are reported according to the STrengthening the Reporting of OBservational studies in Epidemiology (STROBE) recommendations.

### 2.2. Statistical Analysis

A descriptive statistical analysis was performed for clinical features, and data were expressed as number of total and/or percentage or mean ± standard deviation (SD). Some variables were not available for all records, and these were excluded for percentage calculation. Comparison amongst groups at univariate analysis was performed by Chi-squared test. Multivariate logistic regression analyses were used to identify variables related to the dependent variables of interest (any type of anaemia, pernicious anaemia, and iron deficiency anaemia) by including as cofactors all demographic, haematological, and clinical variables of interests. For the logistic regression model, the overall model fit was considered (significant difference of the null model −2 Log Likelihood and the full model −2 Log Likelihood by Chi-Squared test), and the Hosmer and Lemeshow test, a statistical test for goodness of fit for the logistic regression model, was performed, with a *p*-value < 0.05 indicating poor fit and a *p*-value > 0.05 indicating a good logistic regression model fit. Each logistic regression model was run by using the methods “Enter”, “Forward”, “Backward”, and “Stepwise” to enter the independent variables into the model. Variables were removed if *p* > 0.1. Associations of cofactors with the independent variable were taken into consideration when statistical significance was present in all models and were expressed by odds ratio (OR) and 95% confidence intervals (95% CI). The Chi-squared test was performed between age groups and gender, and within age groups. Two-tailed *p* values less than 0.05 were considered statistically significant. Statistical analyses were performed with a dedicated software (MedCalc Software, Mariakerke, Belgium, version 12.7.8).

## 3. Results

### 3.1. Demographic, Clinical, and Histopathological Characteristics

**Figure 1:**
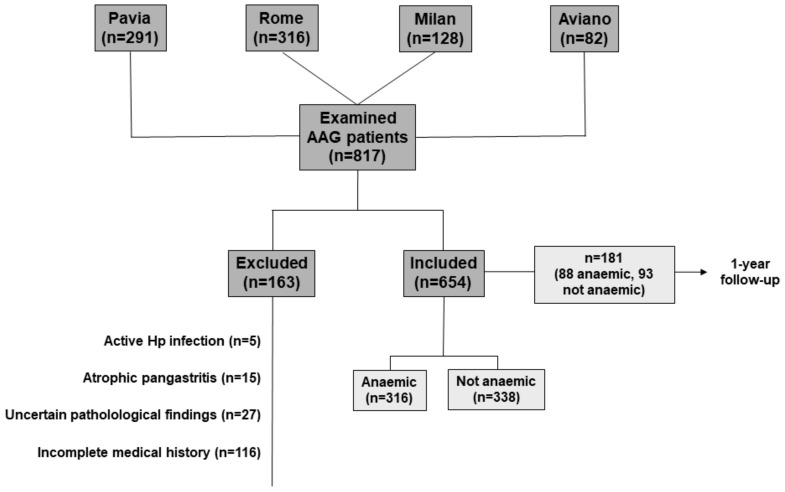
Flowchart of the study. Patients were excluded as per study design. Of the 654 included patients, cell blood count data of 181 (88 anaemic, 93 not anaemic) patients at one-year follow-up were reported. Abbreviations: AAG, autoimmune atrophic gastritis; Hp, *H. pylori*.

A computerised database with records of 817 patients was initially set up (Figure 1). After a careful review, 163 patients were excluded because they did not match the inclusion criteria or because of missing data. Thus, the total number of enrolled patients was 654 (mean age 59.2 ± 13.8 years, female: male ratio = 2.3:1), for whom at least the baseline complete cell blood count and clinical presentation were available. Other variables were not available for all patients, hence statistical analyses were preformed after exclusion of patients with missing data, as detailed in the tables. Of these, 287 patients (43.9%) were from Pavia, 268 (41.0%) from Rome, 74 (11.3%) from Milan, and 25 (3.8%) from Aviano. Relevant demographic and clinical data are reported in Table 2. Indeed, more than one sign, symptom, or alteration may have occurred in the same patient. Female gender was predominant, and 470 patients (71.8%) were aged > 50 years. No statistical difference emerged between male and female gender when plotted according to age groups. 295 patients (46.8%) suffered from another concomitant autoimmune disease, which was autoimmune thyroid disease in most cases (251 patients; 39.9%). Other less common autoimmune diseases included type 1 diabetes, vitiligo, psoriasis, coeliac disease, and primary biliary cholangitis. According to the Sydney–Houston criteria, the mean severity score of corpus atrophy (information available for 549/654 patients) was 2.6 ± 0.6. Operative Link on Gastritis Assessment (OLGA) scores 1 and 2 were observed in 52 (9.4%) and 497 (90.5%) patients, respectively. Gastric corpus intestinal metaplasia was present in 437 (79.6%) patients, with a mean severity score of 1.2 ± 0.8, whereas Operative Link on Gastric Intestinal Metaplasia Assessment (OLGIM) scores 1, 2, and 3 were present in 208 (37.8%), 225 (40.9%), and 4 (0.7%) patients, respectively. Pseudopyloric metaplasia was seen in 409 (62.5%) patients.

### 3.2. Haematological Alterations 

**Figure 2:**
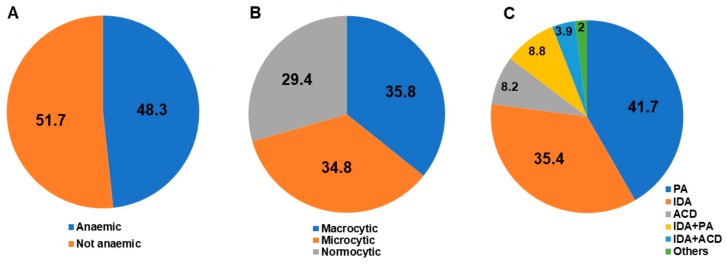
The study population includes 654 adult patients suffering from autoimmune atrophic gastritis. The figure shows the proportion of patients with and without anaemia (**A**), the distribution of mean corpuscular volume (MCV) in anaemic patients (**B**), and subtypes of anaemia (**C**). The category “Others” includes chronic kidney failure and neoplastic diseases. Abbreviations: ACD, anaemia of chronic disease; IDA, iron deficiency anaemia; PA, pernicious anaemia.

Haematological alterations were the leading cause of AAG diagnosis (Table 2). Overall, the mean haemoglobin level was 116 ± 26 g/L, the mean MCV was 90.7 ± 16 fL, and the mean RDW was 15.9% ± 4.3%. The mean platelet count was 236.9 ± 95.4/microliter, while thrombocytopenia or thrombocytosis were observed in 92 (15.2%) and in 13 (2.1%) patients, respectively. Anaemia, which was observed in 316 patients (48.3%; mean age 60.1 ± 15.8 years, F:M ratio = 2.3:1), was mild (haemoglobin ≥ 95 g/L) in 194 patients (61.4%), moderate (haemoglobin 80–94 g/L) in 52 patients (16.4%), and severe (haemoglobin < 80 g/L) in 70 patients (22.2%). Figure 2 reports the proportion of patients with and without anaemia (A), according to MCV (B), and subtypes of anaemia (C). Patients with anaemia had a significantly higher prevalence of MCV abnormalities, anisocytosis, vitamin B12 and folate deficiency, and thrombocytopenia in comparison to non-anaemic patients. Table 3 reports all relevant demographic, clinical, haematological, and histological variables of AAG patients according to the presence or absence of anaemia (any type). With regards to non-anaemic patients, MCV and RDW alterations were present in 76 (23.9%) and in 113 (39.1%) cases, respectively. A significantly higher prevalence of gastrointestinal symptoms, autoimmunity, and treated H. pylori infection was also seen in non-anaemic compared to anaemic patients (Table 3). 

### 3.3. Micronutrient Deficiencies

Regardless of anaemia, vitamin B12 deficiency was observed in 291 (50.1%) patients and iron deficiency in 327 (57.8%) patients. Combined vitamin B12 and iron deficiency was present in 136 (25.1%) cases. Folate deficiency was seen in 34 (9.3%) patients and was associated with vitamin B12 or iron deficiency in 17 (2.6%) and 4 (0.6%) cases, respectively. Folate deficiency anaemia, without concurrent iron or vitamin B12 deficiency, was not observed at all. Patients with anaemia had a significantly higher prevalence of vitamin B12 and folate deficiency compared to non-anaemic patients, while iron deficiency was equally distributed between the two groups (Table 3). Hyperhomocysteinaemia was equally distributed between anaemic and non-anaemic patients (50.6% versus 51.7; *p* = 0.778). Regarding non-anaemic patients, at least one micronutrient deficiency was present in 224/338 (66.3%) cases.

### 3.4. Haematological Alterations with Regard to Gender 

In all AAG patients, while normocytosis was equally distributed between genders, microcytosis occurred more frequently in females (24.0% versus 15.5%; *p* = 0.0237) and macrocytosis more frequently in males (36.4% versus 20.3%; *p* < 0.0001). Pernicious anaemia (27.1% versus 12.4%; *p* = 0.0015) and vitamin B12 deficiency without anaemia (56.3% versus 46.8%; *p* = 0.0448) were more frequent in males, while iron deficiency anaemia (16.9% versus 10.0%; *p* = 0.0393) and iron deficiency without anaemia (64.4% versus 40.9%) were more frequent in females. Combined pernicious and iron deficiency anaemia was similarly distributed between genders (13.3% vs. 9.1%; *p* = 0.1970). Thrombocytopenia was nearly twofold more common in males compared to females (21.2% vs. 12.7%; *p* = 0.0108). Finally, folate deficiency (16% vs. 6.5%; *p* = 0.007) and hyperhomocysteinaemia (63.2% vs. 45.9%; *p* = 0.0011) were both more frequent in males. 

### 3.5. Haematological Alterations with Regard to Age (as a Continuous Variable)

Overall, AAG patients with microcytosis were younger (54.7 ± 15.5 versus 61.1 ± 14.5 years; *p* < 0.0001), while those with macrocytosis were older (63.2 ± 13.8 versus 58.5 ± 15.1; *p* = 0.0008). Patients with iron deficiency anaemia (56.8 ± 16.6 versus 60.2 ± 14.6 years; *p* = 0.0436) and combined pernicious and iron deficiency anaemia (55.9 ± 15.3 versus 60.2 ± 14.9 years; *p* = 0.0211) were younger, while patients with pernicious anaemia were older (63.0 ± 14.6 versus 58.9 ± 14.9 years; *p* = 0.0144). Finally, hyperhomocysteinaemia (61.3 ± 15.1 versus 57.6 ± 15.2 years; *p* = 0.0101) was more commonly observed in older patients. 

### 3.6. Clinical Predictors of Anaemia

Table 4 shows multivariable results for all possible factors associated with anaemia (any type), pernicious anaemia, and iron deficiency anaemia. At logistic regression analysis, anaemia (any type) was significantly associated with anisocytosis, thrombocytopenia, absence of gastrointestinal symptoms, and no proton pump inhibitor use. Moreover, iron deficiency anaemia was significantly associated with anisocytosis and absence of thrombocytopenia, while pernicious anaemia was significantly associated with anisocytosis, thrombocytopenia, and no history of *H. pylori* infection.

### 3.7. Follow-Up of Anaemia

**Figure 3:**
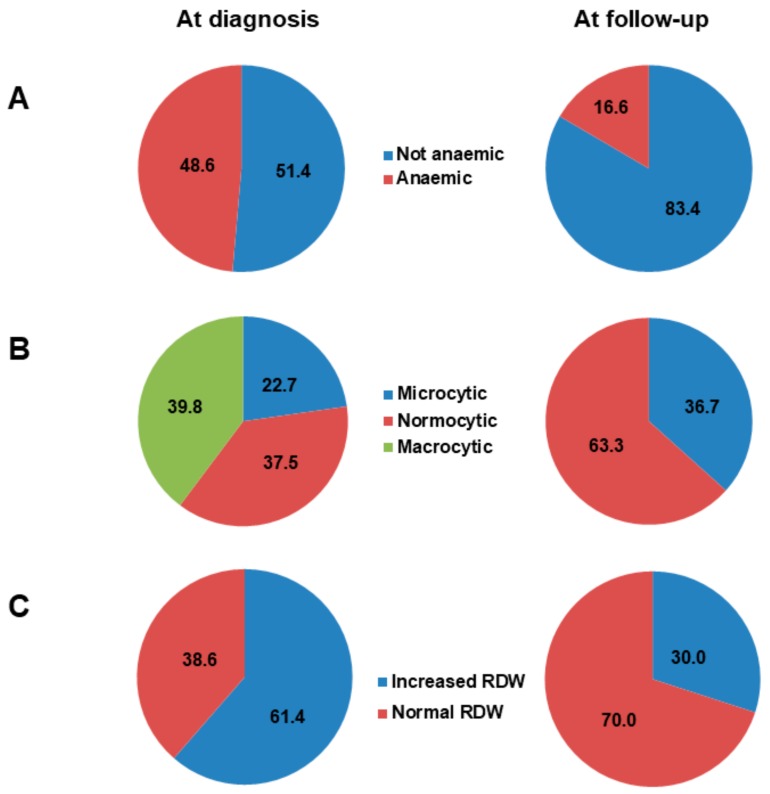
Sub-analysis of a cohort of 181 patients (mean age 59.2 ± 14.6 years, female: male ratio = 2.7:1) who were followed-up for at least one year. The figure shows the proportion of patients with and without anaemia at diagnosis and at the one-year follow-up (**A**), the distribution of mean corpuscular volume (MCV) in anaemic patients at diagnosis and at the one-year follow-up (**B**), and the distribution of red cell distribution width (RDW) at diagnosis and at the one-year follow-up (**C**). Abbreviations: RDW, red cell distribution width.

In a sub-cohort of 181 AAG patients (mean age 59.2 ± 14.6 years, F:M ratio = 2.7:1), we compared cell blood count at diagnosis and after treatment of anaemia according to current recommendations [5,7,28]. All patients received parenteral supplementation of vitamin B12 and oral folic acid, and serum levels were normal at one year in all cases. Oral daily iron supplementation with different commercially available formulations was the first-line treatment of iron deficiency, while only three patients required an intravenous formulation. As shown in Figure 3A, a significantly (*p* < 0.0001) lower proportion of anaemic patients was observed at the one-year follow-up in comparison to baseline. Remarkably, stratification of anaemic patients according to MCV significantly (*p* = 0.003) changed after therapy, with complete recovery of macrocytosis (Figure 3B). The proportion of anaemic patients with anisocytosis significantly (*p* < 0.0001) decreased after treatment (Figure 3C). Regarding patients with persistent microcytic anaemia only, this was mild in all cases, with depletion of iron stores despite oral supplementation.

## 4. Discussion

Here we have reported red blood cell alterations in a large cohort of patients with histologically proven AAG at the time of diagnosis. Roughly half of AAG patients were anaemic, being pernicious anaemia and iron deficiency anaemia the most prevalent subtypes, with gender- and age-related differences. Anisocytosis was the strongest predictor of anaemia. Of note, a considerable proportion of non-anaemic AAG patients showed micronutrient deficiencies. 

Recently, the clinical burden of anaemia in a number of immune-mediated gastrointestinal disorders, including inflammatory bowel disease and coeliac disease, has gained a great interest [29,30]. With regards to AAG, only one study specifically focused on patterns of anaemia and haematologic alterations in biopsy unproven AAG, and only patients who were referred for iron deficiency anaemia or vitamin B12 deficiency were enrolled [6]. In this study, some cases of chronic gastritis without atrophy and active H. pylori infection were also included. Moreover, the prevalence of anaemia and related haematologic alterations in AAG have been described in other series, but only as a part of the general clinical description (see Table 1). Our study reveals that haematologic alterations are the leading reasons for AAG diagnosis. Recently, we have also shown that AAG diagnosis could be delayed partly due to the lack of physician awareness, especially among gastroenterologists, of all possible clinical presentations of this condition [18]. Compared to the presence of overt anaemia, diagnostic delay was longer for scant haematologic alterations (<12 months versus 12–24 months), including isolated microcytosis and macrocytosis. Hence, these subtle alterations should not be overlooked and should prompt further investigations, starting from AAG serological tests [31]. According to previous reports [7,28,32], AAG should be ruled out in all cases of vitamin B12 deficiency and in case of unexplained iron deficiency anaemia. Our data provide further background for supporting this strategy. 

Half of AAG patients were anaemic at the time of diagnosis. The crude prevalence of pernicious anaemia was similar to that of iron deficiency anaemia but was different when adjusted for gender and age. Particularly, iron deficiency anaemia was more common in younger female patients, whereas pernicious anaemia was more common in older male patients. These findings may be explained by the slow evolution of AAG, that may take several years to progress [1,33,34]. It is reasonable to assume that iron deficiency characterises earlier disease stages, in which hypo-achlorhydria causes iron malabsorption, even if additional unknown immunological factors may have a role. Conversely, vitamin B12 is stored in large amounts and it takes years for pernicious anaemia to develop. 

From a pathophysiological point of view, there should be no differences between female and male AAG patients with regard to iron absorption. However, a possible explanation could be that iron deficiency is more likely in women of reproductive age, due to menstrual blood loss. In premenopausal women, menstrual flow per se is a possible confounding factor, frequently causing iron deficiency anaemia, and postmenopausal status is often able to reverse mild anaemia [35]. Data regarding menstrual cycle and menopause were not available, hence no firm conclusion can be drawn in this regard. 

The inclusion of patients at different time points of AAG natural history may also explain other findings. Unexpectedly, there seems to be no correlation between anaemia and severity of histological damage, according to both OLGA and OLGIM classifications. Even patients showing severe gastric corpus atrophy might have been enrolled at any time after development of the histological changes. This could also explain the high prevalence of vitamin B12 deficiency, iron deficiency, or a combination of both in patients without anaemia, possibly reflecting the enrolment at an early disease stage. However, mechanisms other than gastric mucosal atrophy could explain our findings. For example, the role of other factors, including hypo-achlorhydria, bacterial overgrowth, impairment of iron metabolism, and immunological alterations secondary to AAG still needs to be elucidated. 

At multivariate analysis, anisocytosis was the strongest predictor of anaemia. Interestingly, increased RDW has been associated with a higher all-cause mortality rate in the general population [36]. The association of anaemia with the lack of gastrointestinal symptoms is less clear but could reflect a longer diagnostic delay [18] that may favour the occurrence of anaemia. Instead, the association of pernicious anaemia with the lack of H. pylori infection may underlie the pathogenic difference between AAG and post-infectious atrophic gastritis. However, there is still ongoing debate in this regard, with contrasting results [37], and more studies are needed to ascertain this hypothesis. To sum up these results, in clinical practice, the sole presence of anisocytosis should raise the suspicion of AAG, while no other clear predictors were found. 

According to the one-year follow-up, most cases of anaemia recovered. Particularly, pernicious anaemia recovered in all cases, while a proportion of patients still had iron deficiency anaemia. These data seem to indicate that iron deficiency anaemia might take more time to recover and oral iron supplementation therapy might fail to restore iron storage adequately. 

Some limitations of the study must be mentioned. First, patients might have been diagnosed at different times of AAG natural course, thus affecting the prevalence of haematologic alterations. Also, transferrin and laboratory inflammatory markers were not available in our series. Further, our study did not evaluate the clinical impact of anaemia in terms of disabling symptoms, including fatigue and malaise. Nonetheless, this is the largest cohort of AAG patients that has been described so far, showing that overt anaemia and subtle red blood cell alterations are frequent and are the leading cause of AAG diagnosis.

## 5. Conclusions

More attention should be paid to scant haematologic alterations, most commonly due to vitamin B12 and iron deficiency. Prompt micronutrient supplementation is warranted in AAG patients, as could prevent (or revert) the development of anaemia. Optimal treatment of iron deficiency anaemia still needs to be defined.

## Figures and Tables

**Table 1:** Main studies reporting the prevalence of anaemia in adult patients with autoimmune atrophic gastritis (AAG).

Authors	Year	Setting	Patients (n)	Enrolled at AAG Diagnosis	Mean Age (years)	Overall Anaemia (%)	PA (%)	IDA (%)	Major Findings
Burman et al. [11]	1991	Internal medicine	86	Yes	56	80.2	80.2	NA	High PCA levels associated with PA
Hersko et al. [6]	2006	Haematology	160	NS	59	75.0	18.1	51.8	Microcytic anaemia more common in younger age
Lahner et al. [12]	2009	Gastroenterology	165	Yes	54	94.5	49.0	45.5	PCA and anti-intrinsic factor antibodies yield 73% accuracy PA diagnosis
Miceli et al. [4]	2012	Gastroenterology	99	Yes	58	59.6	59.6	0	PA was the most common cause of AAG diagnosis
Lahner et al. [13]	2015	Gastroenterology	83	Yes	59	51.8	51.8	NA	A genetic variant of transcobalamin 2 was related to PA
Zhang et al. [14]	2017	Gastroenterology	275	Yes	61	19.3	5.4	8.4	No clear gender differences; microcytic anaemia more common in younger age
Kalkan et al. [15]	2017	Gastroenterology	355	NS	57	30.1	30.1	NA	PA more common in older patients
Carabotti et al. [16]	2017	Gastroenterology	379	Yes	55	88.4	53.6	34.8	PA more frequent in patients without gastrointestinal symptoms
Villanacci et al. [17]	2017	Pathology	138	NS	48	54.7	25.0	29.7	IDA more common than PA
Lenti et al. [18]	2019	Gastroenterology	291	Yes	60	49.5	23.4	11.7	Isolated MCV alterations associated with greater AAG diagnostic delay

Abbreviations: IDA, iron deficiency anaemia; MCV, mean corpuscular volume; NA, not assessed; NS, not specified; PA, pernicious anaemia; PCA, anti-parietal cell antibody. Studies exploring atrophic gastritis prior to *H. pylori* discovery were not included. Mean age refers to anaemic patients only, whenever this datum is available.

**Table 2:** Main demographic and clinical features of patients with autoimmune atrophic gastritis (AAG).

Demographic Features	
Total AAG patients, *n*	654
Females, *n* (%)	459 (70.2)
Age (years), median (range)	61 (18–88)
Main clinical presentation at diagnosis, *n* (%)	
Haematological	368 (56.3)
Gastrointestinal	109 (16.7)
Neuropsychiatric	105 (16.1)
Endocrinological	23 (3.5)
Miscellaneous	53 (8.1)
Other relevant clinical information, *n* (%)	
Current or past smoking	246 (37.7)
Cardiovascular disease	384 (58.7)
Autoimmune comorbidities	305 (46.7)
Neuropsychiatric disorders	180 (27.5)
Family history of AAG	55 (8.4)
Family history of gastric cancer	16 (2.4)
Proton pump inhibitor use prior to diagnosis	237 (36.3)
Previously treated *H. pylori* infection	97 (14.8)

**Table 3:** Clinical, haematological, and histological variables of patients with autoimmune atrophic gastritis (AAG) according to the presence or absence of anaemia (any type).

	Presence of Anaemia	Absence of Anaemia	*p*-Value
Female gender	220/459 (47.9)	239/459 (52.1)	0.8916
Male gender	96/195 (49.2)	99/195 (50.8)	0.9563
Age (years) mean ± SD	60.1 ± 15.8	59.3 ± 13.9	0.5061
*Age groups (years)*			
≥80	31 (10.0)	10 (3.0)	Trend 0.5463
70–79	74 (23.9)	82 (24.8)	
60–69	64 (20.6)	89 (26.8)	
50–59	47 (15.2)	73 (22.0)	
40–49	61 (19.7)	41 (12.3)	
30–39	24 (7.7)	32 (9.6)	
≤29	9 (2.9)	5 (1.5)	
Current or past smoking	103/300 (34.3)	114/324 (35.2)	0.8894
Gastrointestinal symptoms	32/302 (10.6)	75/310 (24.2)	<0.0001
Endocrinological disorders	7/302 (2.3)	16/312 (5.1)	0.1050
Cardiovascular disease	178/301 (59.1)	188/317 (59.3)	0.9674
Neuropsychiatric disorders	80/297 (26.9)	90/313 (28.7)	0.6816
Autoimmune comorbidities	130/305 (42.6)	165/325 (50.8)	0.0491
Autoimmune thyroid disease	108/303 (35.6)	143/325 (44.0)	0.0399
Family history of AAG	19/296 (6.4)	33/314 (10.5)	0.1136
Family history of gastric cancer	6/296 (2.0)	9/314 (2.9)	0.6838
PPI use prior to diagnosis	63/216 (29.2)	97/223 (43.5)	0.0025
Previous *H. pylori* infection	26/276 (9.4)	61/311 (19.6)	0.0008
Macrocytosis	108/303 (35.6)	48/317 (15.1)	<0.0001
Microcytosis	105/303 (34.6)	28/317 (8.8)	<0.0001
Normocytosis	90/303 (29.7)	241/317 (76.0)	<0.0001
Vitamin B_12_ deficiency	167/278 (60.1)	124/303 (40.9)	<0.0001
Iron deficiency	163/280 (58.2)	164/286 (57.3)	0.9007
Folate deficiency	24/165 (14.5)	10/201 (4.9)	0.0031
Vitamin B_12_ and iron deficiency	72/263 (27.4)	64/279 (22.9)	0.2749
Increased RDW	227/277 (81.9)	113/289 (39.1)	<0.0001
Thrombocytopenia	69/294 (23.5)	23/310 (7.4)	<0.0001
Thrombocytosis	6/294 (2.0)	7/310 (2.2)	0.9231
Intestinal metaplasia	246/310 (79.3)	258/332 (77.7)	0.6814
Pseudopyloric metaplasia	196/301 (65.1)	194/317 (61.2)	0.3547
OLGA 1 OLGA 2	22/269 (8.2) 247/269 (91.8)	30/280 (10.7) 250/280 (89.3)	Trend 0.3851
OLGIM 0 OLGIM 1 OLGIM 2 OLGIM 3	56/269 (20.8) 102/269 (37.9) 111/269 (41.3) 0 (0)	56/280 (20.0) 106/280 (37.9) 114/280 (40.7) 4/280 (1.4)	Trend 0.6349

Abbreviations: OLGA, Operative Link for Gastritis Assessment; OLGIM, Operative Link on Gastric Intestinal Metaplasia Assessment; PPI, proton pump inhibitor; RDW, red blood cell distribution width; SD, standard deviation. Percentages were calculated after exclusion of patients with missing data. *p* < 0.0001 were more frequent in females. Combined pernicious and iron deficiency anaemia was similarly distributed between genders (13.3% versus 9.1%; *p* = 0.1970). Thrombocytopenia was nearly two-fold more common in males compared to females (21.2% versus 12.7%; *p* = 0.0108). Finally, folate deficiency (16% versus 6.5%; *p* = 0.007) and hyperhomocysteinaemia (63.2% versus 45.9%; *p* = 0.0011) were both more frequent in males.

**Table 4:** Multivariate logistic regression analysis for variables associated with anaemia (any type), iron deficiency anaemia, and pernicious anaemia in patients with autoimmune atrophic gastritis (AAG).

Variables	Odds Ratio	95% CI	*p*-Value
Autoimmune comorbidities	0.67	0.2306–1.9532	0.4644
No history of *H. pylori* infection	1.48	0.7288–3.0118	0.2775
Thrombocytopenia	3.27	1.4596–7.3507	0.0040
Increased RDW	10.65	6.1381–18.5012	<0.0001
Absence of gastrointestinal symptoms	2.26	1.1048–4.6312	0.0256
Autoimmune thyroid disease	0.92	0.3127–2.7167	0.8825
Vitamin B12 deficiency	1.09	0.6359–1.8960	0.7371
No PPI use prior to diagnosis	1.98	1.1071–3.5423	0.0213
PCA positivity	0.92	0.4873–1.7438	0.8024
Age < 50 years	0.67	0.3830–1.1922	0.1760
Family history of gastric cancer	0.45	0.0823–2.5594	0.3744
No family history of AAG	1.77	0.7096-4.4185	0.2207
Current or past smoking	0.78	0.4638-1.3122	0.3494
Autoimmune comorbidities	0.51	0.1683-1.5950	0.2517
No history of *H. pylori* infection	1.69	0.8029-3.5970	0.1657
Absence of thrombocytopenia	2.20	1.0789-4.5053	0.0301
Increased RDW	4.83	2.7097-8.6409	<0.0001
Female gender	1.47	0.8227-2.6430	0.1921
PCA positivity	1.39	0.6535-2.9741	0.3900
Age < 50 years	0.66	0.3372-1.3286	0.2511
No family history of AAG	1.26	0.4861-3.3118	0.6266
Current or past smoking	1.13	0.6082-2.1290	0.6860
Autoimmune comorbidities	0.60	0.1775-2.0391	0.4145
No history of *H. pylori* infection	2.98	1.1249-7.9012	0.0280
Thrombocytopenia	3.51	1.7113-7.2044	0.0006
Increased RDW	6.40	3.1210-13.1643	<0.0001
Female gender	0.33	0.38 to 1.42	0.3663

Abbreviations: PCA, anti-parietal cell antibody; RDW, red cell distribution width. The analysis included 549 patients with complete data.
